# Natural Fat Nanoemulsions for Enhanced Optical Coherence Tomography Neuroimaging and Tumor Imaging in the Second Near-Infrared Window

**DOI:** 10.1021/acsnano.4c01204

**Published:** 2024-03-11

**Authors:** Xiaorui Geng, Xiao Liang, Yubin Liu, Yuhao Chen, Bin Xue, Xianyuan Wei, Zhen Yuan

**Affiliations:** † Cancer Center, Faculty of Health Sciences, University of Macau, Taipa, Macau SAR 999078, China; ‡ Centre for Cognitive and Brain Sciences, University of Macau, Taipa, Macau SAR 999078, China; § Department of Biomedical Engineering, Southern University of Science and Technology, Shenzhen, 518055, China; ∥ College of Photonics and Electric Engineering, Fuzhou Normal University, Fuzhou, 350117, China; ⊥ Shenzhen Key Laboratory of Ultraintense Laser and Advanced Material Technology, Center for Advanced Material Diagnostic Technology, and College of Engineering Physics, Shenzhen Technology University, Shenzhen, 518118, China

**Keywords:** optical coherence tomography, angiography, nanoemulsion, tumor imaging, neuroimaging

## Abstract

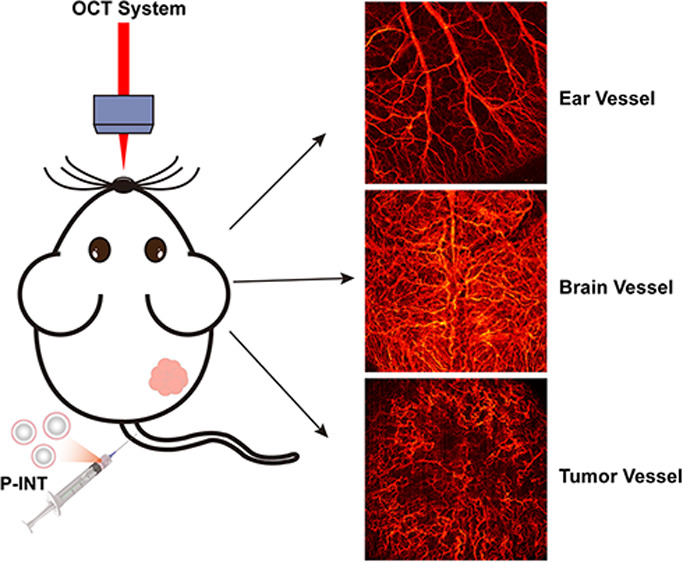

Optical coherence tomography (OCT) imaging mainly uses backscattered light to visualize the structural and functional information on biological tissues. In particular, OCT angiography can not only map the capillary networks but also capture the blood flow in the tissue microenvironment, making it a good candidate for neuroimaging and tumor imaging *in vivo* and in real time. To further improve the detection accuracy of cancer or brain disorders, it is essential to develop a natural and nontoxic contrast agent for enhanced OCT imaging in the second near-infrared (NIR-II) window. In this study, a superior biocompatible and highly scattering NIR-II fat nanoemulsion was constructed to improve OCT imaging contrast and depth for monitoring the vascular network changes of the cerebral cortex or tumor. *In vivo* experimental results demonstrated that a natural fat nanoemulsion can serve as an excellent probe for enhanced OCT neuroimaging and tumor imaging.

## Background

To date, optical coherence tomography (OCT) is able to noninvasively visualize the structural and functional information on biological tissues with high temporal resolution (less than 1 s) and micrometer spatial resolution.^1−6^ OCT has been demonstrated as a robust imaging method for the diagnosis of various diseases in cardiology, dermatology, and ophthalmology.^7−13^ In particular, the use of exogenous contrast is able to improve the contrast of the OCT imaging for the detection of brain diseases and tumors. Interestingly, various OCT contrast agents have been developed with strong optical scattering properties such as dyes, gold and silver nanoparticles, which can specifically bind to the diseased tissues, thereby increasing the contrast between the diseased and normal tissues.^14^ For example, air-filled or oil-filled microbubbles can serve as the positively optical-scattering contrast agent for OCT molecular imaging, whereas gold nanorods or near-infrared (NIR) dyes as negative contrast agents are also used for enhanced OCT imaging.^15−18^ Besides, retroreflective-type Janus microspheres were able to effectively enhance the back-reflecting light for high-accuracy OCT imaging.^19^ Further, multifunctional nanoprobes were constructed for OCT-based multimodality molecular imaging and imaging-guided disease theranostics.^20,21^ However, existing OCT contrast agents were basically constructed at the first NIR (700–900 nm) window. Therefore, it remains a big challenge to develop probes that are responsive to the NIR-II (1000–1700 nm) window for deep imaging with increased maximum permissible exposure.^20^

More importantly, Intralipid (INT) is the FDA approved agent for nutritional supply of patients lacking essential fatty acids.^21^ In addition, INT solution is usually used to produce optical tissue phantoms *in vitro*, whose scattering coefficients can be accessed according to the signal intensity measured by NIR spectroscopy. The main components of INT consist of the soybean oil, lecithin, glycerin, and water, in which the soybean oil and lecithin with high optical scattering properties exhibit the potential to serve as the OCT contrast agent.^22,23^ However, INT is able to be rapidly cleared from the blood circulation system *in vivo*, significantly limiting its efficacy as an OCT theranostics agent.

**Figure 1:**
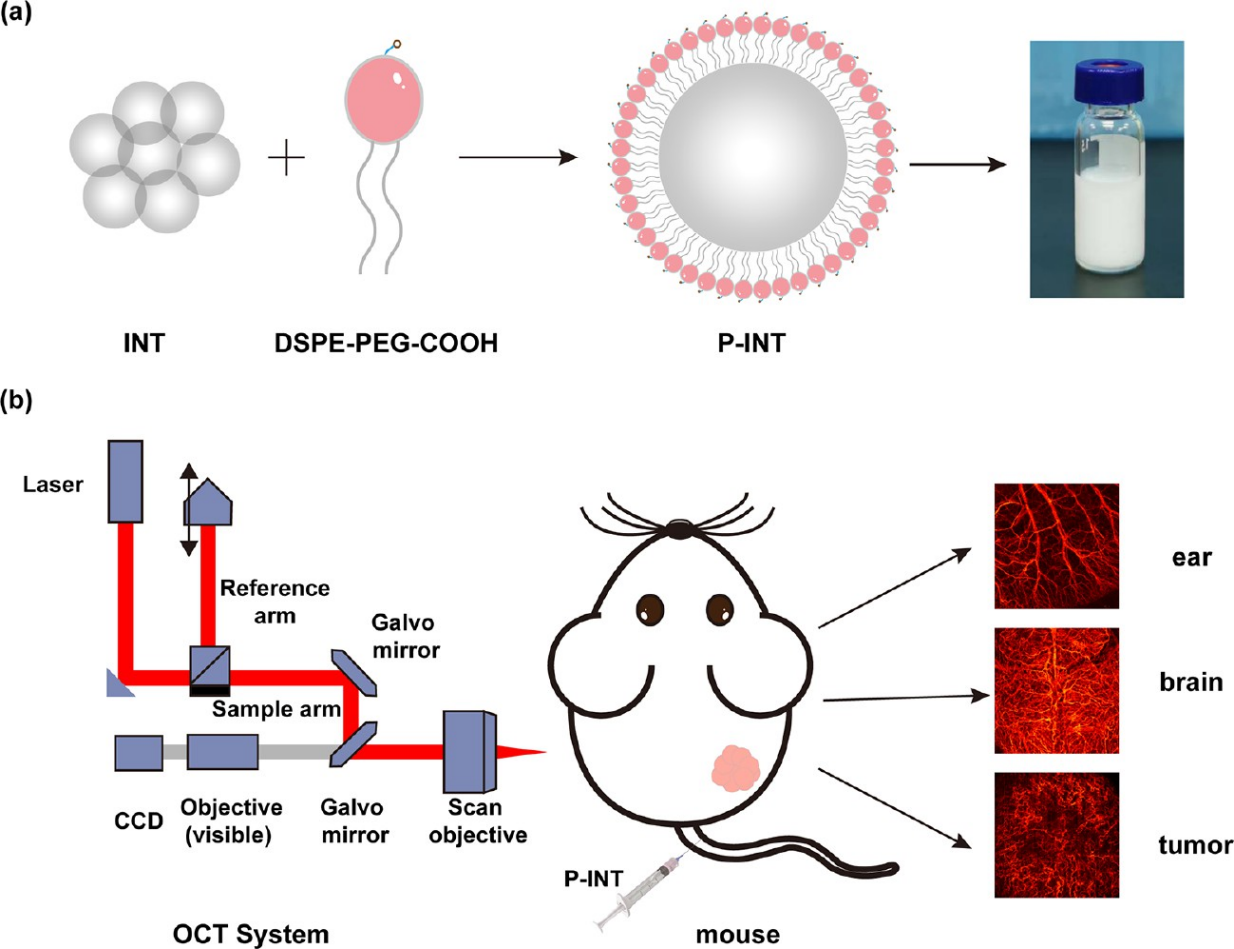
Schematic of the prepared P-INT. Schematic of angiography of the tumor and cerebral cortex by our homemade OCT imaging system after tail-vein injection of P-INT.

In this study, a facile nanoengineering method is demonstrated to modify INT with DSPE-PEG2000-COOH to develop it into a type of fat nanoemulsion (P-INT). *In vitro* and *in vivo* NIR-II OCT imaging tests were also carried out, indicating the enhanced OCT signal generation ability of P-INT (Figure 1). Besides, the biocompatibility analysis and biodistribution *in vivo* further confirmed the zero toxicity and body clearance ability of P-INT. In particular, this natural P-INT showed a high water solubility, prolonged blood circulation time *in vivo*, and enhanced OCT neuroimaging and tumor imaging capabilities in the NIR-II window. Therefore, this pilot study develops highly biocompatible and natural OCT contrast agents, illustrating the extremely high potential of P-INT for future clinical translation.

## Results and Discussion

### Characterization of P-INT

**Figure 2:**
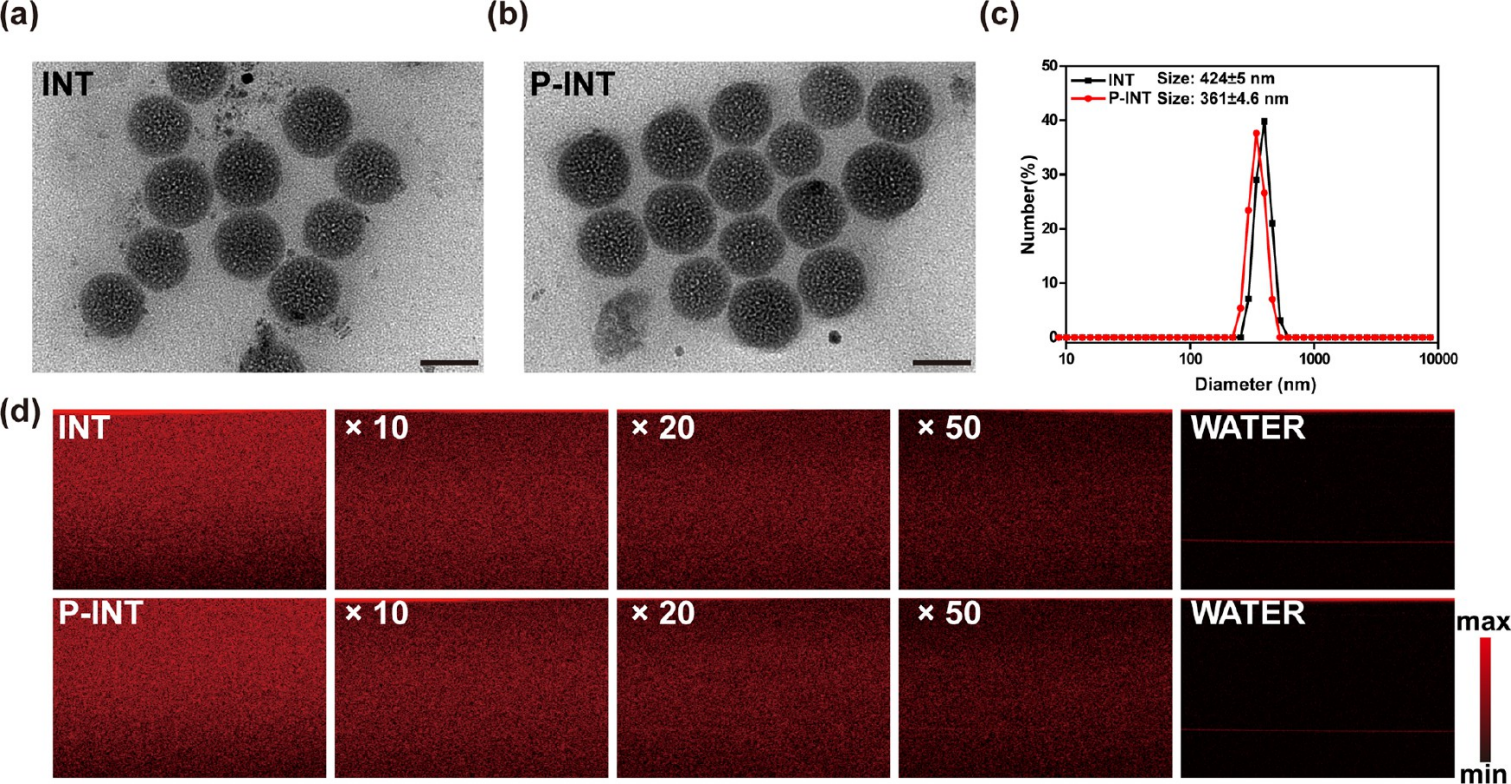
Characterization of natural fat nanoemulsion P-INT. TEM images of INT (a) and P-INT(b); scale bar = 100 nm. (c) Size distributions of INT and P-INT measured by dynamic light scattering. (d) The OCT signal intensity (scattering properties) of INT and P-INT decreased with increased diluted concentrations from 10 to 50 times.

Figure 2a and b display the TEM images of INT and P-INT, respectively. The TEM imaging results demonstrated that the morphologies of INT and P-INT were uniform and round, showing no significant difference. The results of the dynamic light scattering (DLS) showed that the particle size of INT and P-INT was 424 ± 5 nm and 361 ± 4.6 nm (Figure 2c; Figure S1). Additionally, the zeta potentials of INT and P-INT were −34.57 ± 0.42 mV and −38.87 ± 0.55 mV (Figure S2), respectively. Compared to that of INT, the reduced particle size of P-INT is because PEG can increase the solubility and fluidity of INT in aqueous solution.^24^ The zeta potential of P-INT decreased after modification with DSPE-PEG-COOH, possibly due to the negative charge carried by DSPE-PEG-COOH. The absorption and fluorescence spectra analysis indicated that the absorbance and emission of INT and P-INT decreased with increased wavelength, without a noticeable peak in the NIR-II window (Figure S3). Both INT and P-INT showed good stability at pH 5.6–7.4 for 24 h, indicating that P-INT has potential as a contrast agent for tumor imaging (Figure S4). Further, phantom tests were performed to inspect the OCT imaging ability of P-INT. Likewise, the scattering properties of both INT and P-INT decreased with their increased diluted concentrations from 10 to 50 times. The quantitative results illustrated that there is no statistical difference in the scattering properties between INT and P-INT (Figure S5). The modification of DSPE-PEG-COOH was able to enhance the fluidity and stability of INT, showing no significant effect on its scattering properties.

### OCT Angiography Imaging of the Mouse Ear

**Figure 3:**
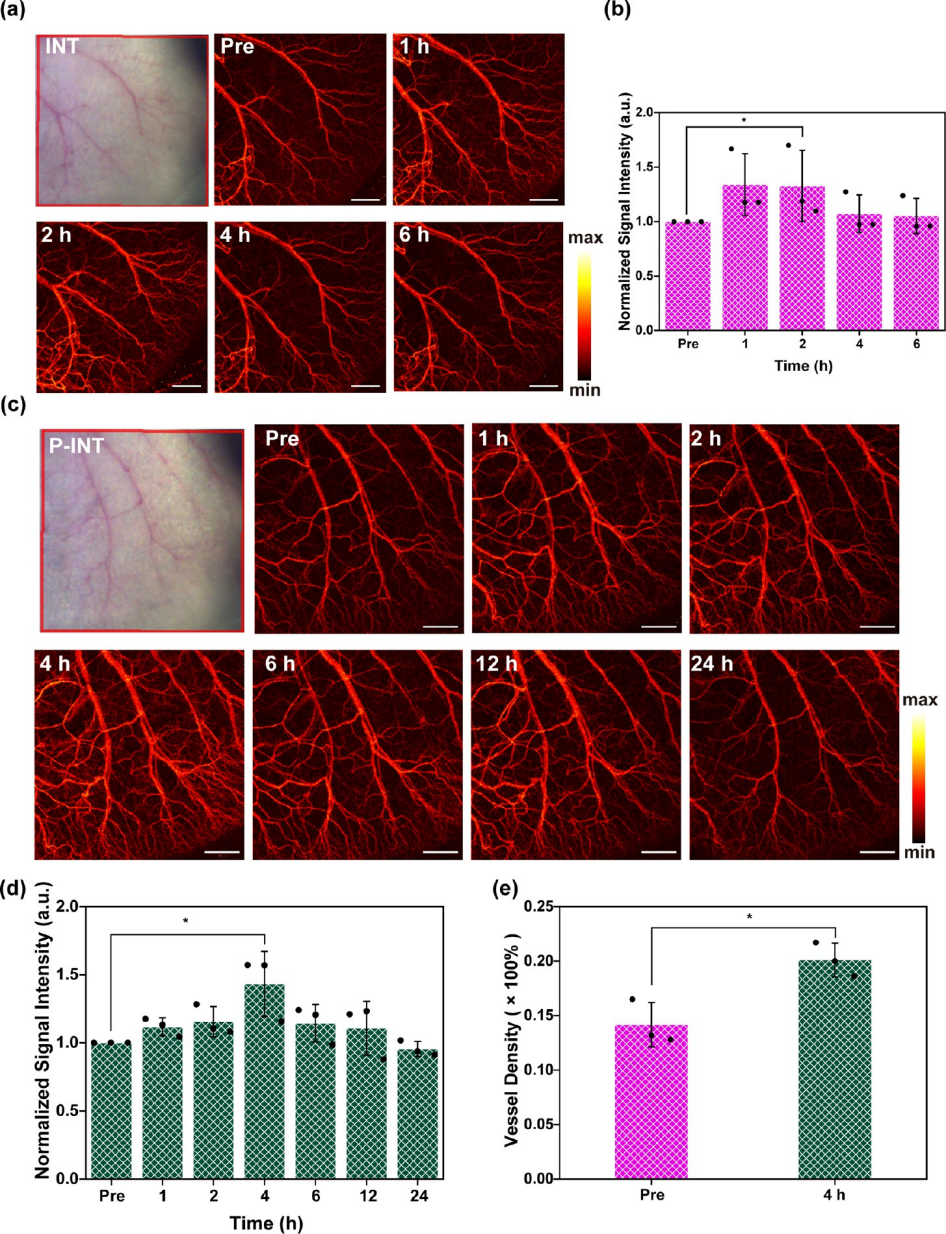
OCT angiography imaging (a) and signal intensity (b) of the mouse ear at different time points after tail-vein injection of INT. Scale bar = 1 mm. OCT angiography imaging (c) and signal intensity (d) of the mouse ear at different time points after tail-vein injection of P-INT. Scale bar = 1 mm. (e) Measured blood vessel density 0 and 4 h postinjection of P-INT.

In order to assess the enhanced OCT imaging capability of P-INT *in vivo*, the vascular structures of the ear site of BALB/c mice were first imaged. Figure 3 demonstrated the captured mouse ears at different time points after tail-vein injections of INT and P-INT. It was discovered that both INT and P-INT can enhance the OCT imaging contrast. However, the OCT signal strength reached a peak at 1 h after tail-vein injection of INT, whereas that of P-INT group reached the peak 4 h postinjection (Figure 3a and c). Interestingly, when the OCT signal intensity reached the peak, they began to decrease, demonstrating that both INT and P-INT had excellent biocompatibility and were able to be easily metabolized from the body *in vivo*.

Meanwhile, PEG modification on P-INT was able to significantly improve the *in vivo* circulation time of INT. Figure 3b and d demonstrate the quantitative analysis of the OCT imaging signal intensity at various time points. It was discovered that the peak of the signal intensity 1 h postinjection of INT was 1.34 ± 0.28 times higher than that of preinjection. By contrast, the peak of signal intensity 4 h postinjection of P-INT was 1.44 ± 0.14 times higher than that of preinjection. In addition, the signal intensity 4 h postinjection of INT value was 1.07 ± 0.17 times higher than that of preinjection. More importantly, it was discovered from Figure 3e that due to the improved OCT imaging contrast, the blood vessel density after the injection of P-INT was significantly increased as compared to that before injection.

### Enhanced OCT Neuroimaging

Morphological analysis of the vascular structural changes in the cerebral cortex is crucial for the diagnosis and treatment of various brain disorders. The penetration depth is the advantage of OCT in the NIR-II window. For NIR-II OCT imaging, the depth can be up to 3 mm (Figure S6a,b), although the imaging contrast decreased with increased depth. Blood flow was also calculated for different depths (0–3 mm). OCT imaging results at different depths showed that the vascular network was the densest at the depth of 0.5–1 mm. However, it is very hard to capture the accurate blood flow with a depth over 1.5 mm (Figure S6b,c).

**Figure 4:**
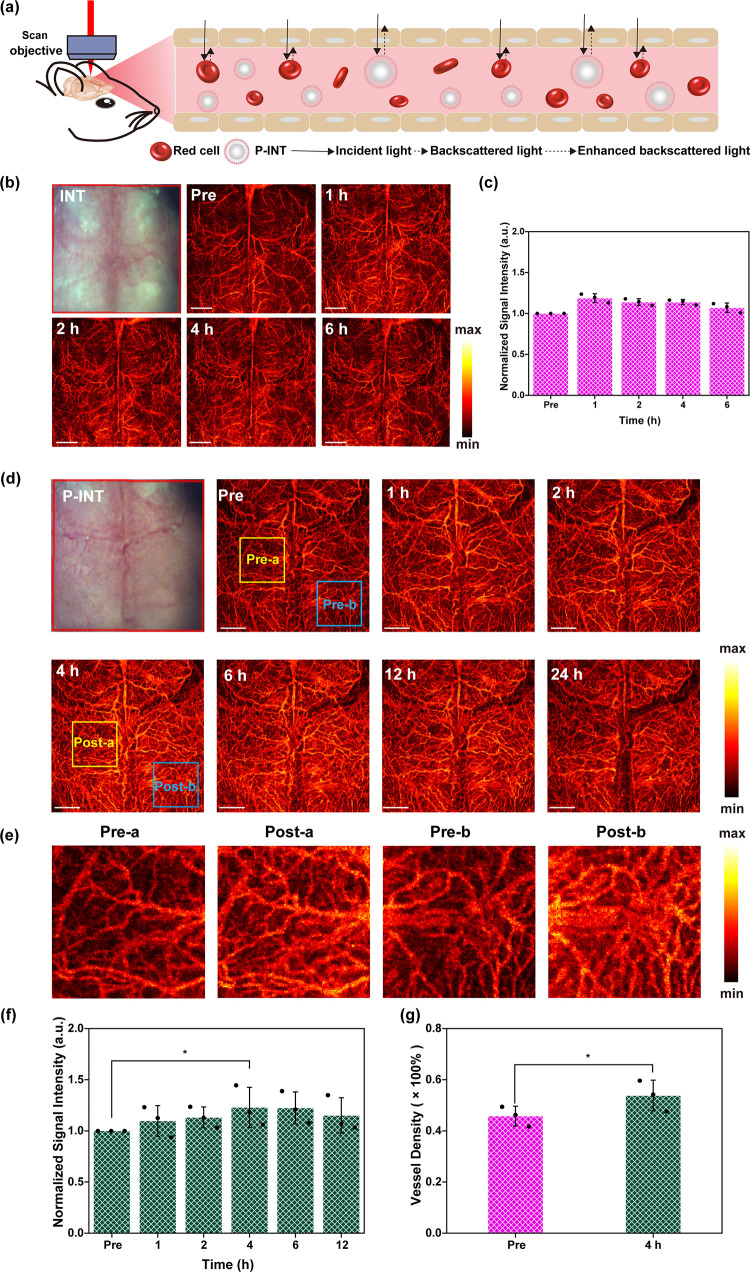
P-INT-enhanced OCT neuroimaging. (a) Schematic of increased backscattering ability of P-INT in brain vascular structures. (b) OCT vascular imaging of the brain at different time points after tail-vein injection of INT. Scale bar = 1 mm. (c) Signal intensity of brain vascular imaging after tail-vein injection of INT. The data were not statistically different between the groups. (d) OCT imaging of brain blood vessels at different time points after tail-vein injection of P-INT. Scale bar = 1 mm. (e) Local enlarged images (regions a and b) of brain vascular structures before (pre) and after (post) the injection (0 h: Pre-a/Pre-b, 4 h: Post-a/Post-b). (f, g) Quantification of signal intensity (f) and density (g) of brain blood vessels at different time points after tail-vein injection of P-INT.

In this study, the use of OCT neuroimaging was carried out to inspect the imaging accuracy of mouse brain when P-INT served as the contrast agent. As displayed in Figure 4, the injection of P-INT (10 mg/kg) via the tail vein improved both the OCT imaging contrast (vascular signal) and resolution. This improvement was due to the increased scattering signal of P-INT distributed in the blood vessels (Figure 4a). Consistent with the results of ear angiography, INT was able to enhance the brain angiography imaging, and the OCT signal intensity reached a peak between 1 and 2 h postinjection (Figure 4b,c). In particular, the cerebral vascular signals began to increase after the injection of P-INT until reaching the peak 4 h postinjection (Figure 4d,e) and then began to decrease although were still higher than that of preinjection (Figure 4f). More importantly, the resolution was also significantly improved, since more cerebral vascular structures were detected in detail, demonstrating increased blood vessel density (Figure 4g). The cerebral vascular signals at different depths were enhanced 4 h postinjection of P-INT, resulting in a significantly denser vascular network. In particular, the signals were significantly improved at depths of 1–2 mm and 2–3 mm, which might be beneficial for imaging deep tissues. The cross-section of the brain showed significant increase in signal intensity 4 h postinjection as compared to that before injection (Figure S7). Likewise, increased vascular signal was associated with increased blood flow in the brain, which can serve as a neural marker to detect brain diseases or cognitive neural functions. Generally speaking, neural activity will result in an increase in blood flow and blood oxygen. Such hemodynamic or metabolic activity can be detected by fMRI.^25−28^ Here, we show that OCT also has the potential to measure this signal in small animals. Therefore, P-INT as a natural and multifunctional contrast can improve the sensitivity of OCT neuroimaging for the diagnosis of various cerebral vascular-related diseases.

### Enhanced OCT Tumor Imaging

In addition, OCT tumor imaging was also conducted to show the advantages of P-INT for cancer detection. In particular, a melanoma tumor-bearing mouse model was developed to detect the vascular information change associated with tumor microenvironments. It is noted that due to the strong optical absorption of melanin, conventional optical imaging methods such as photoacoustic and fluorescence imaging have challenges in detecting the blood vessels of melanoma tumors. By contrast, an optical scattering-based contrast agent such as P-INT might show the potential to detect the vasculature network changes in melanoma tumor microenvironments by OCT. First, the B16 melanoma-bearing mouse model and 4T1 breast tumor-bearing mouse model were established to study the effect of melanin on optical imaging. For B16 melanoma-bearing mice, the OCT imaging provided clear visualization of the morphology and density of melanoma blood vessels, while the intratumoral blood vessel signal was obscured by the presence of skin surface melanin during photoacoustic microscope imaging. The morphology of the tumor vessels can be detected by both imaging techniques in mouse breast tumor (Figure S8).

**Figure 5:**
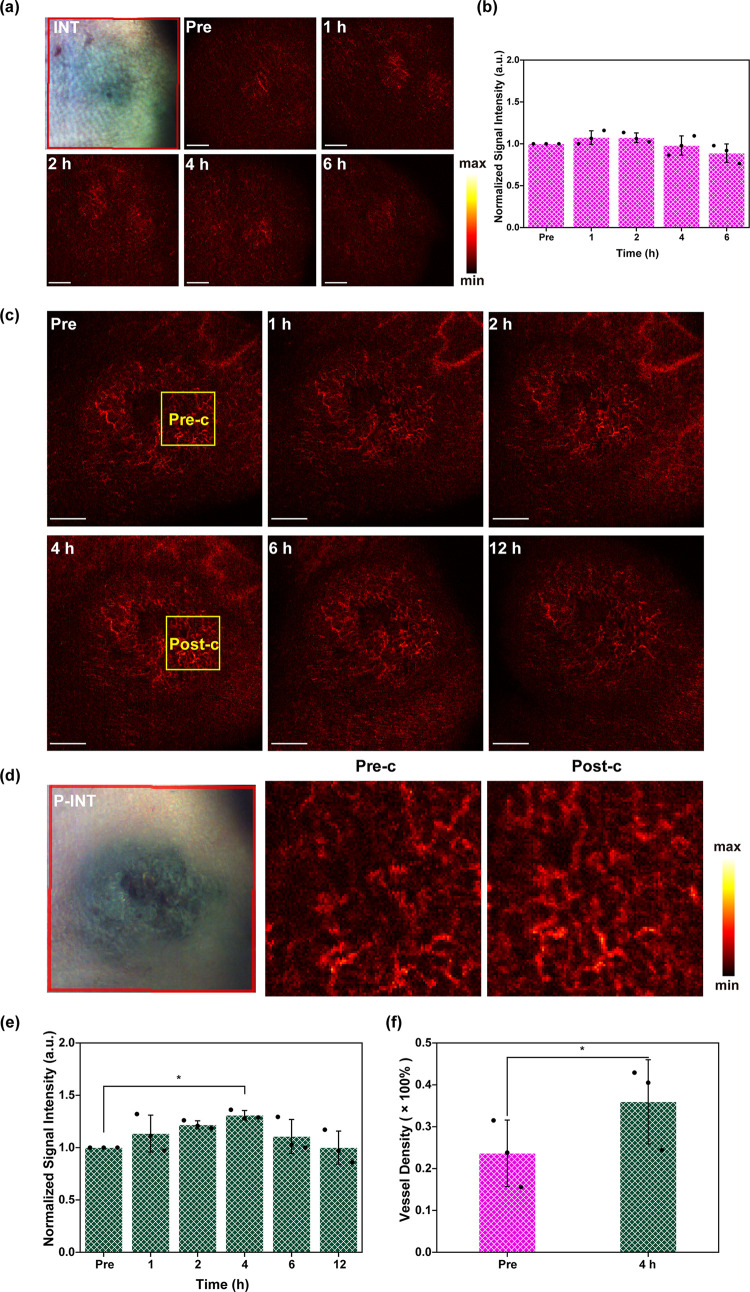
OCT tumor angiography imaging (a) and signal intensity (b) of melanoma-bearing mice at different time points after tail-vein injection of INT. Scale bar = 1 mm. (c) OCT tumor angiography imaging at different time points after tail-vein injection of P-INT. Scale bar = 1 mm. (d) Local enlarged imaging region c before (Pre) and after (Post) injection of P-INT (0 H: Pre-c, 4 H: Post-c). The left of (b) is the photograph of melanoma tumor. (e, f) Quantification of signal intensity (e) and density (f) of tumor blood vessels at different time points after tail-vein injection of P-INT.

In addition, OCT tumor images and vascular signal intensities were acquired at different time points after tail-vein injection of P-INT or INT into melanoma-bearing mice (Figure 5a–d). The tumor blood vessel signal intensity was enhanced and reached a peak 1 h postinjection of INT (Figure 5a,b). Further, the tumor blood vessel signals began to increase after the injection until reaching a peak 4 h postinjection of P-INT (Figure 5c–e). As plotted in Figure 5d, both the imaging contrast and the resolution were improved after P-INT was used as the contrast agent. In particular, it was discovered that the blood vessel density 4 h postinjection of P-INT was 1.52 times higher than that of preinjection (Figure 5f). Therefore, OCT tumor imaging is able to offer an effective diagnostic tool for tumor vessel microenvironments.

### Monitoring Tumor Angiogenesis by Enhanced OCT Imaging

It is also important to monitor tumor angiogenesis by visualizing the morphology changes of tumor blood vessels with a high temporospatial resolution. In this study, NIR-II OCT imaging was performed to monitor the tumor vessel microenvironment changes of melanoma tumor *in vivo* with high spatial resolution in real time.

**Figure 6:**
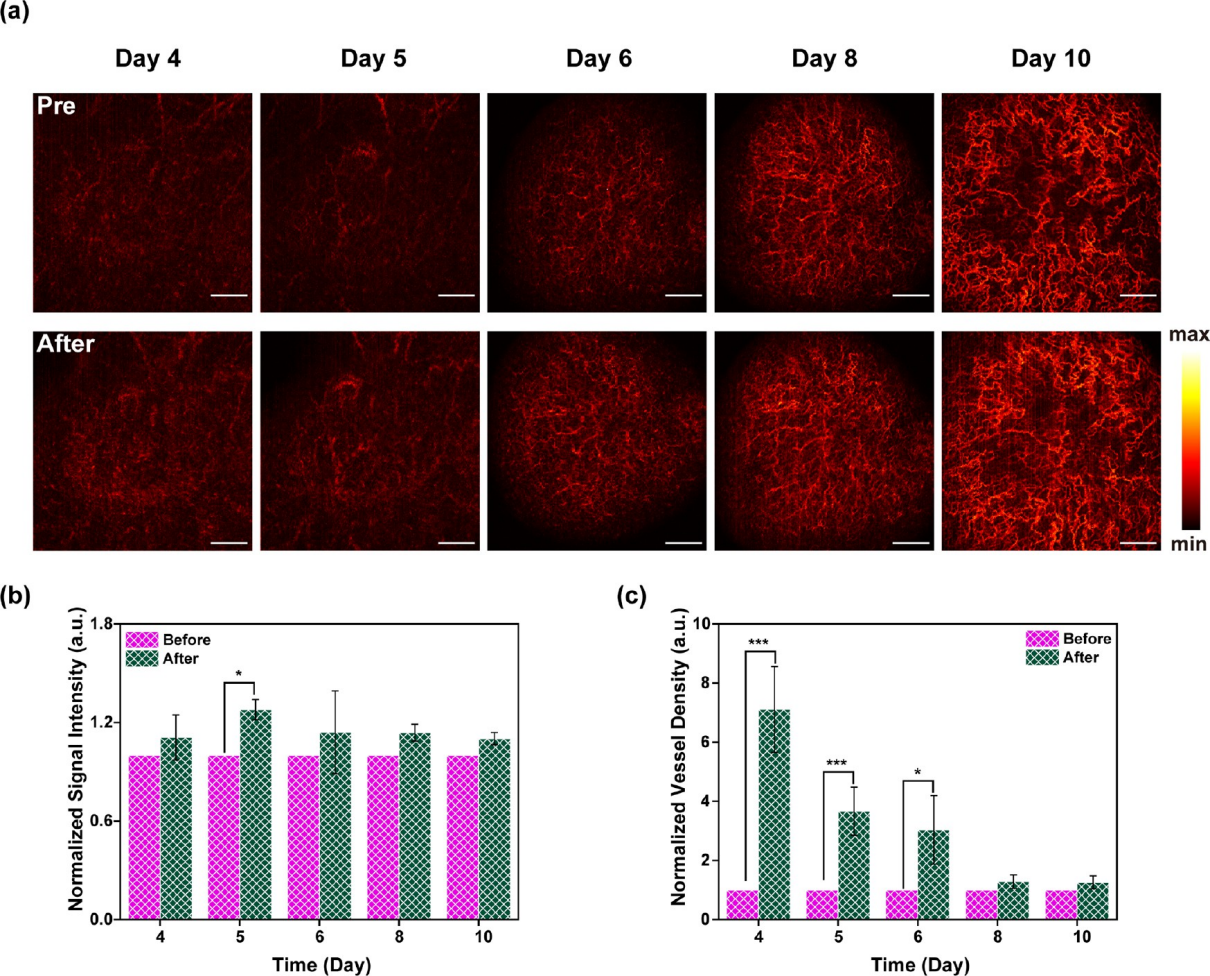
(a) Monitoring of tumor angiogenesis without (first row) and with (second row) the tail-vein injection of P-INT. Scale bar = 1 mm. (b, c) Quantification of signal intensity (b) and density (c) of tumor blood vessels at different time points after tail-vein injection of P-INT.

Living cancer cells were injected into the superficial areas to develop a melanoma tumor-bearing mouse model. OCT imaging was then carried out 4 days postinjection of the tumor cells to monitor the vascular structure changes in tumor microenvironments. Interestingly, it was discovered that the blood vessels at the tumor site increased with increased days of tumor growth (Figure 6a). In particular, both the blood vessel density and blood vessel signal intensity were significantly increased when P-INT was used as a contrast agent as compared to those without enhancement (Figure 6b,c). All the imaging results demonstrated that P-INT can serve as a good contrast agent for monitoring tumor angiogenesis.

### Biosafety of P-INT

**Figure 7:**
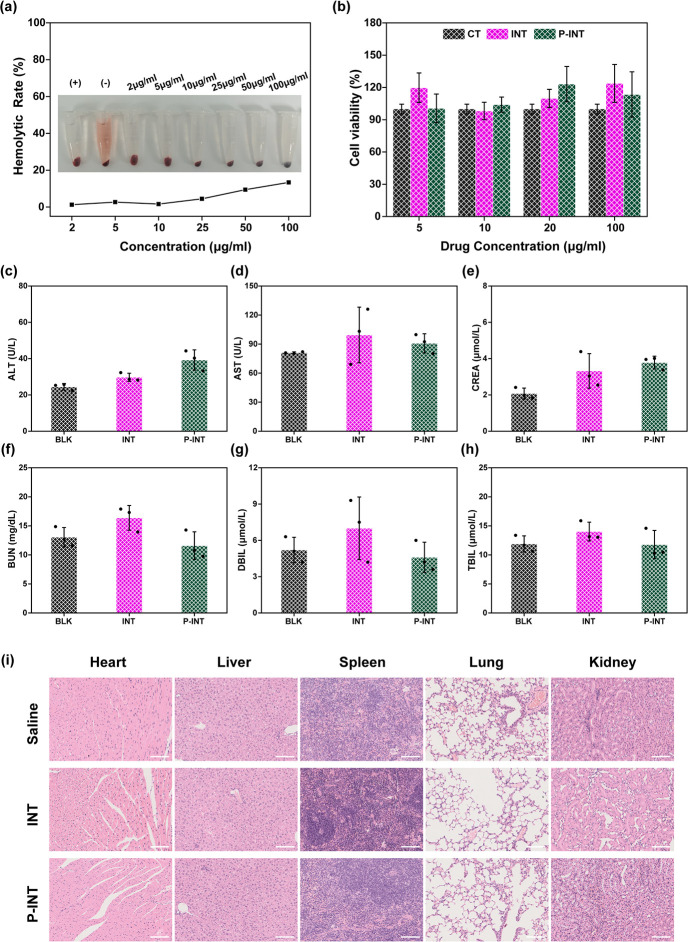
Biosafety of P-INT. (a) Hemolytic efficiency of P-INT at various concentrations. Positive control (+): ddH_2_O; negative control (−): PBS. (b) The cell viability of 293T cells treated with different concentrations of INT and P-INT for 12 h. (c–h) Biochemical analysis of mouse blood after 7 days of different treatments (injection of saline, INT, and P-INT) (*n* = 3). (i) Representative H&E-stained images of the major organs from mice after 7 days of different treatments (injection of saline, INT, and P-INT) (*n* = 3). Scale bar = 100 μm.

The biosafety of contrast agents plays a crucial role for their potential clinical translation. Therefore, the biocompatibility of P-INT was inspected here including the hemolysis, cytotoxicity, and *in vivo* toxicity. The red blood cells of mice were utilized for hemolysis analysis. It was found that at doses of up to 100 μg/mL P-INT still had excellent blood safety with a hemolysis rate of less than 15% (Figure 7a). After incubation with different concentrations of INT or P-INT for 12 h, the cell viability of 293T cells (human renal epithelial cell) was measured. The results indicated that nanoemulsion increased the cell proliferation rate (Figure 7b). Even with the addition of laser irradiation, INT or P-INT treatment did not affect the activity of 293T cells (Figure S9).

The *in vivo* metabolic results showed that the metabolic time of P-INT was longer than that of INT (Figure S10). Further, the mice were randomly divided into three groups (*n* = 3) and received different treatments: (1) saline (BLK), (2) INT (10 mg/kg), and (3) P-INT (10 mg/kg). Seven days and 6 weeks after injection, the mouse blood and organs were collected for blood biochemical tests and pathological analysis. It was found that the biochemical indexes were within the normal range (Figure 7c–h, Figure S11). Additionally, the organs for H&E sectioning showed no significant difference compared to those of the normal mouse group (Figure 7i, Figure S12). Therefore, P-INT has the potential for clinical translation due to its excellent biocompatibility.

### Comparison of P-INT with Other OCT Imaging Contrast Agents

**Figure 8:**
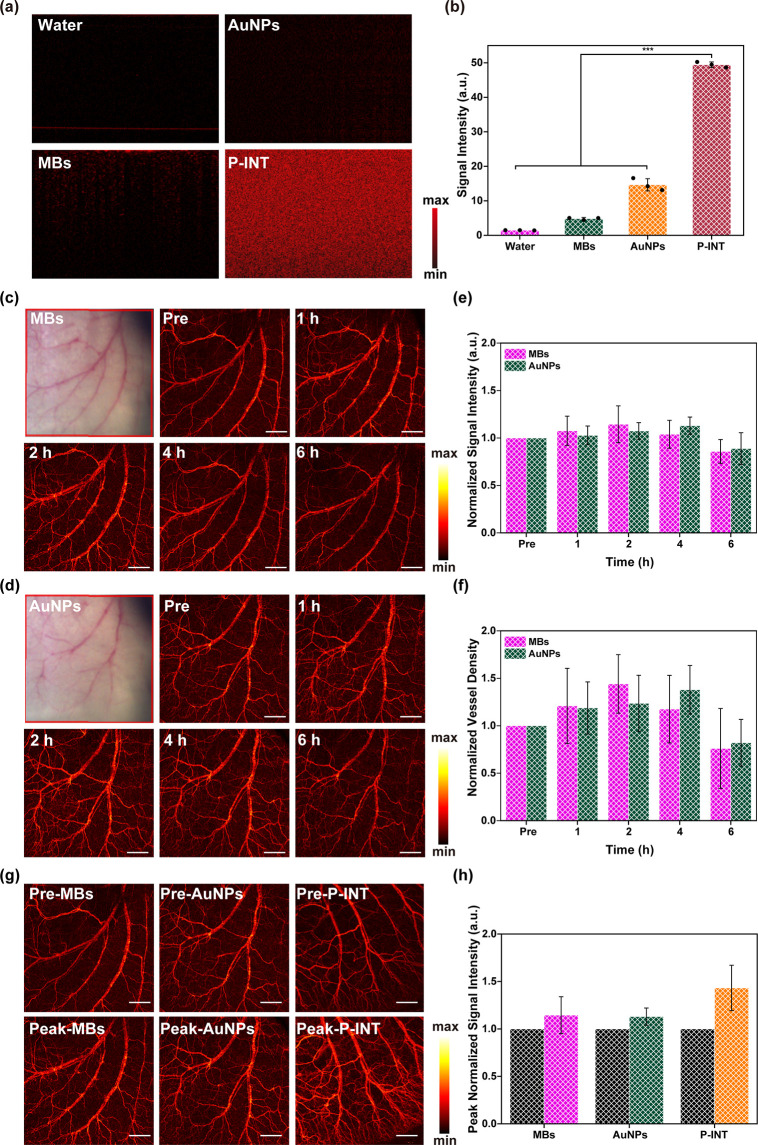
Comparison of P-INT with other contrast agents. (a, b) Scattering properties (a) and quantitative analysis (b) of water, AuNPs, MBs, and P-INT. (c, d) OCT angiography imaging of the mouse ear at different time points after tail-vein injection of MBs and AuNPs. Scale bar = 1 mm. (e) The signal intensity of the mouse ear at different time points after tail-vein injection of MBs and AuNPs. (f) The vessel density of the mouse ear at different time points after tail-vein injection of MBs and AuNPs. (g, h) OCT angiography imaging and signal intensity of the mouse ear at the peak time point after tail-vein injection of MBs, AuNPs, and P-INT. Scale bar = 1 mm.

Recently a number of OCT contrast agents in the NIR-II window have been developed for *in vivo* imaging. For example, Assadi et al. proposed the use of microbubbles (MBs) for inspecting tissue hemodynamics using enhanced OCT angiography.^29^ Si et al. demonstrated that gold nanobipyramids can serve as OCT multiplexing contrast agents, enabling NIR-II high-resolution imaging of two distinct lymphatic flows occurring simultaneously from different drainage basins into the same lymph node in live mice.^30^ In addition, Nguyen et al. reported an ultrapure chain-like gold nanoparticle (AuNP) for OCT eye imaging in rabbits.^31^ Here, we compared the OCT imaging ability among P-INT, MBs, and AuNPs. First, optical scattering analysis demonstrated that P-INT exhibited the strongest scattering property among the three contrast agents (Figure 8a,b). Further *in vivo* tests showed that MBs were able to enhance the OCT imaging signal in the mouse ear, which reached a peak 2 h postinjection. Likewise, AuNPs exhibited the same phenomena, demonstrating that the OCT signal reached a peak 4 h postinjection (Figure 8c–f). Both MBs and AuNPs were capable of improving the detection of blood vessels. However, the enhanced imaging effect of P-INT was significantly superior to those of MBs and AuNPs (Figure 8g,h).

To inspect the impact of different PEG chain lengths on blood circulation time of P-INT, DSPE-PEG1000-COOH and DSPE-PEG5000-COOH were respectively used to prepare short-chain PEG-INT (SP-INT) and long chain PEG-INT (LP-INT). We discovered that the scattering properties of SP-INT and LP-INT were similar to those of P-INT and INT (Figure S13). When incubated with 293T cells, SP-INT and LP-INT demonstrated good biocompatibility (Figure S14). The quantitative results of signal intensity in the OCT images of the mouse ear showed that the peak time of SP-INT was between 2 and 4 h and the peak time of LP-INT was at 4 h, which showed no significant difference from those of P-INT. Therefore, the difference in PEG chain length showed no significant effect on the blood circulation time of P-INT (Figure S15). In summary, the enhanced OCT angiography imaging ability of P-INT was superior to other OCT contrast agents such as MBs and AuNPs. And P-INT also exhibited the larger potential for clinical translation in consideration of excellent biocompatibility.

## Conclusions

In summary, we have developed a natural nanoemulsion (P-INT) for enhanced OCT imaging. It was discovered that P-INT can significantly improve both the OCT imaging contrast and resolution for the detection and monitoring of brain diseases and tumors. Therefore, our study provides a potential platform to monitor the morphology changes of vascular structures of brain and tumors with high potential for future clinical translation.

## Methods

### Preparation of P-INT

A 40 mg amount of DSPE-PEG2000-COOH (Xi’an Ruixi Biological Technology Co., Ltd.), 2 mL of 20% INT (Sichuan Kelun Pharmaceutical Co., Ltd.), 6 mL of ultrapure water, and 1 mL of tetrahydrofuran (Anaqua) were mixed and sonicated for 30 min at 4 °C. The solution was vigorously stirred for 24 h and then dialyzed (MWCO: 10 kDa) to remove the organic solvent. To analyze the effect of different lengths of PEG chains on the scattering properties, DSPE-PEG2000-COOH was replaced with DSPE-PEG1000-COOH and DSPE-PEG5000-COOH (Xi’an Ruixi Biological Technology Co., Ltd.) to prepare and obtain SP-INT and LP-INT as controls.

### Characterization of P-INT

The morphology of INT and P-INT was respectively imaged by transmission electron microscope (TEM). INT (10 μL) and P-INT (10 μL) were dropped on a copper mesh, dried, and tested on the TEM. The diameters of nanoemulsion particles were measured by DLS (NanoZS 90, Malvern, USA). Absorption and emission spectra of INT and P-INT were detected by a UV spectrophotometer and fluorescence spectrometer. The scattering properties of different concentrations of INT and P-INT were detected by our homemade spectral domain optical coherence tomography (SD-OCT) imaging system. Scattered signal intensity of INT and P-INT was obtained from ImageJ software.

### Scattering Properties of Microbubbles and Gold Nanoparticles

Microbubbles (SonoVue) were purchased from Bracco. Gold nanoparticles were synthesized using the well-established sodium citrate reduction method.^32^ A 50 mL amount of deionized water and 1 mL of 1% HAuCl_4_ solution were mixed in a three-necked flask and heated to 100 °C. Then 0.8 mL of 5% sodium citrate solution was added to the flask. A 500 μL amount of MB and AuNP were added in 24-well plates, respectively, and the scattering properties of the solutions were collected using OCT imaging in 2D mode. The signal intensity was obtained from ImageJ measurements.

### Animal Studies

The female BALB/c and C57 mice (6–8 weeks old) purchased from Vital River Laboratory Animal Technology Co (Beijing, China) were used to establish a tumor-bearing mouse model and monitor vascular information. A 100 μL B16 cell suspension (2 × 10^7^ cell/mL) was injected into the right leg of the C57 mice. A 100 μL 4T1 cell suspension (2 × 10^7^ cell/mL) was injected into the right leg of the BALB/c mice. Before the OCT imaging, mice were anesthetized by 5% isoflurane gas. After *in vivo* experiments, all mice were euthanized by carbon dioxide asphyxiation. All animal experiments were approved by the Institutional Animal Care and Use Committee (IACUC) of the University of Macau.

### OCT Imaging System and OR-PAM Imaging System

For our homemade SD-OCT imaging system, the light source is a single superluminescent diode with a central wavelength at 1325 nm and spectral bandwidth over 100 nm. The axial resolution was 12 μm, while the lateral resolution was 13 μm.^19^ The SD-OCT was able to offer depth-resolved and cross-sectional imaging of a small animal in real time. Data collection and image reconstruction were carried out by using a high-performance computer. For comparison with OCT imaging, the tumor’s internal vascular structures were also monitored by using a home-built optical-resolution photoacoustic microscopy (OR-PAM) system. The imaging system parameters are listed here: laser wavelength = 532 nm; lateral resolution = 10 μm; field of view = 10 mm; pulse repetition frequency = 50 kHz; and acquisition time = 20 s.

### *In Vivo* Ear OCT Angiography

Eighteen BALB/c mice were randomly divided into six groups: INT (10 mg/kg), P-INT (10 mg/kg), AuNP (20 mg/kg), microbubbles (SonoVue, 22.5 mg/kg), SP-INT (10 mg/kg), and LP-INT (10 mg/kg). Mouse ears were then depilated for the OCT imaging. Meanwhile, the OCT images were collected at different time points after intravenous injection of contrast agent. To calculate the vascular density, the reconstructed images were first binarized, and then the pixels with a value of 1 were summated to derive *V*_1_. Subsequently, the total number of pixels was quantified to yield the *V*_2_. The vascular density (*D*) can be calculated as *D* = *V*_1_/*V*_2_.

### *In Vivo* Brain OCT Angiography

Six C57 mice were randomly divided into two groups: INT (10 mg/kg) and P-INT (10 mg/kg). The scalps of mice need to be processed to expose the brain. OCT images were collected at different time points.

### *In Vivo* Tumor OCT Angiography

Six melanoma-bearing C57 mice with tumor volumes of about 200 mm^3^ were respectively injected with INT (10 mg/kg) and P-INT (10 mg/kg). OCT images were collected at different time points. At the fourth, fifth, sixth, eighth, and 10th days after the subcutaneous injection of the tumor cell suspension, OCT images were collected at 4 h after the intravenous injection of P-INT (10 mg/kg).

### Biosafety of P-INT

The biosafety of P-INT was evaluated in terms of its hemolytic, cytotoxic, and *in vivo* toxicity. Erythrocytes were collected and incubated with varying doses of P-INT, then centrifuged at 37 °C for 2 h. Pure water was used as a positive control, and PBS was used as a negative control. The supernatants were collected, and their OD at 540 nm was measured.
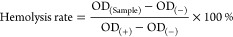


To ensure cell safety, 293T cells were inoculated into 96-well plates. After 12 h, the cells were replaced with serum-free media containing varying concentrations of INT and P-INT. The cells were then incubated for an additional 12 h, and cell viability was assessed using a CCK-8 kit to determine the cytotoxicity of INT and P-INT on 293T cells. To test the biocompatibility of light on cells, 293T cells were incubated with varying concentrations of INT and P-INT for 12 h. The cells were then treated with laser irradiation in an imaging system for 1, 3, 5, and 10 min to observe the cytotoxicity of the laser combined with INT and P-INT.

At the animal level, female C57 mice were randomly divided into three groups: the control group (treated with saline), the INT group (treated with 10 mg/kg), and the P-INT group (treated with 10 mg/kg). Blood samples were collected from each group for biochemical analysis at 7 days and 6 weeks after injection. Concurrently, major organs were extracted from mice in different groups and stained with H&E to evaluate the organ toxicity of P-INT. Samples collected at 7 days were used for short-term *in vivo* safety assessment, while those collected at 6 weeks were used for long-term biocompatibility assessment.

### Biosafety of SP-INT and LP-INT

The viability of 293T cells was used to assess the biosafety of SP-INT and LP-INT at varying concentrations. The procedure followed was as follows: 293T cells were collected and inoculated into 96-well plates. After 12 h, the cells were replaced with serum-free medium containing different concentrations of SP-INT and LP-INT and cultured for an additional 12 h. The viability of the cells was measured by using the CCK-8 kit to assess the cytotoxicity of SP-INT and LP-INT on 293T cells.

The experimental design aimed to detect the blood half-life of INT and P-INT using fluorescent dye labeling. To prepare the samples, 100 μL of Cy5 solution (500 μg/mL) was mixed with 5 mL of INT and P-INT, respectively, using ultrasonic mixing for 30 min. The mixtures were incubated at 50 °C for 24 h before being dialyzed (MWCO: 10 kDa) to remove free dye. The INT and P-INT labeled with Cy5 were injected into the tail vein of C57 mice. Blood was collected from the mouse orbital venous plexus at pre, 5 min, 15 min, 30 min, 1 h, 2 h, 4 h, 6 h, 12 h, and 24 h time points. The blood samples were then scanned by IVIS with an excitation wavelength of 710 nm and an emission wavelength of 760 nm. The images were acquired through automatic exposure, and the blood was analyzed using quantitative fluorescence analysis and the instrument’s software.

### Statistical Analysis

The experimental data were presented as mean ± SD. Statistical significance between different groups was assessed by independent samples *t* test (***P < 0.001; **P < 0.01; *P < 0.05). The confidence interval was 95%. All statistical analyses were analyzed by SPSS 26.0 software.

## Supporting Information Available

Size distribution, zeta potential, absorption and emission spectra, size stability of INT and P-INT, quantitative analysis of signal intensity, penetration depth of OCT, the effect of melanin on OCT imaging and OR-PAM imaging, biocompatibility, quantification of total fluorescence of blood, biochemical analysis, scattering properties of INT, P-INT, SP-INT, and LP-INT, OCT angiography imaging of SP-INT and LP-INT (PDF)
