# Supplementary material for: Natural Fat Nanoemulsions for Enhanced Optical Coherence Tomography Neuroimaging and Tumor Imaging in the Second Near-Infrared Window
Source: ACS Nano. 2024 Mar 11;18(12):9187–98. doi: 10.1021/acsnano.4c01204 (PMC10976961; doi:10.1021/acsnano.4c01204)
Supplement: Supplementary file 1 — nn4c01204_si_001.pdf [file nn4c01204_si_001.pdf]

Supporting Information for

Natural Fat Nanoemulsions for Enhanced Optical Coherence Tomography Neuroimaging and Tumor Imaging in the Second Near Infrared Window

Xiaorui Geng[1,2,#], Xiao Liang[1,2,3,#], Yubin Liu[4,#], Yuhao Chen[1,2], Bin Xue[1,2,5], Xianyuan Wei[1,2], Zhen Yuan[1,2,*]

[1]Cancer Center, Faculty of Health Sciences, University of Macau, Taipa, Macau SAR, 999078, China

[2]Centre for Cognitive and Brain Sciences, University of Macau, Taipa, Macau SAR, 999078, China

[3]Department of Biomedical Engineering, Southern University of Science and Technology, Shenzhen, 518055, China

[4] College Photonics and Electric Engineering, Fuzhou Normal University, Fuzhou, 350117, China

[5]Shenzhen Key Laboratory of Ultraintense Laser and Advanced Material Technology, Center for Advanced Material Diagnostic Technology, and College of Engineering Physics, Shenzhen Technology University, Shenzhen, 518118, China

[*] Corresponding author: zhenyuan@um.edu.mo; [#] represents equal contribution to this work

Supplementary Figures

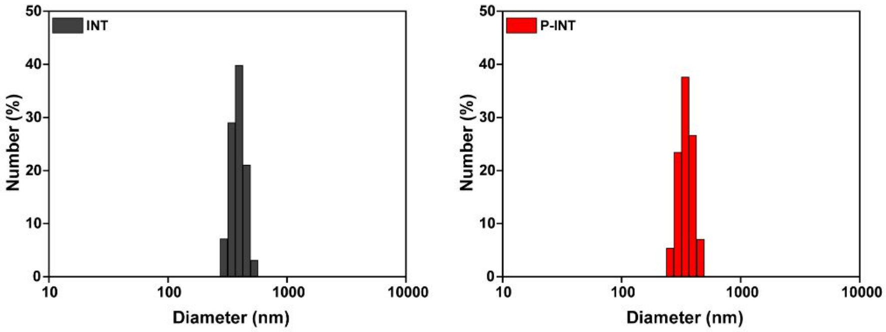

Fig. S1 The size distribution of INT and P-INT.

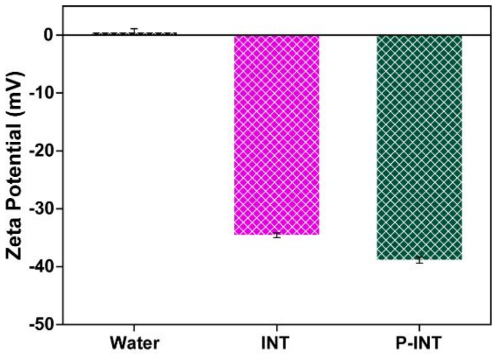

Fig. S2 The Zeta potential of water, INT and P-INT.

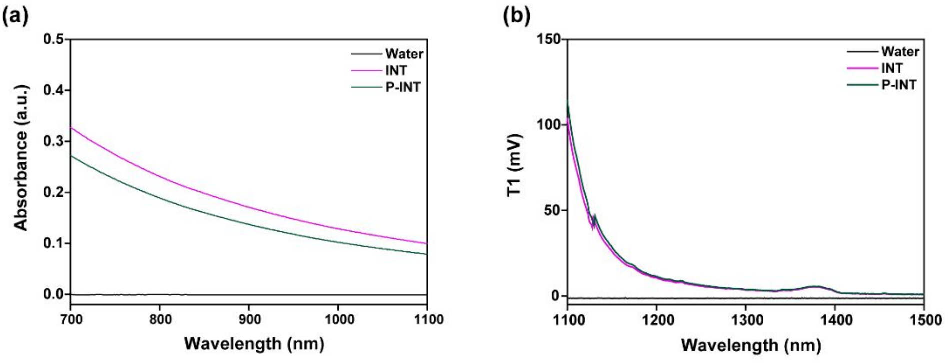

Fig. S3 The absorption and emission spectra of water, INT and P-INT in the NIR-II window.

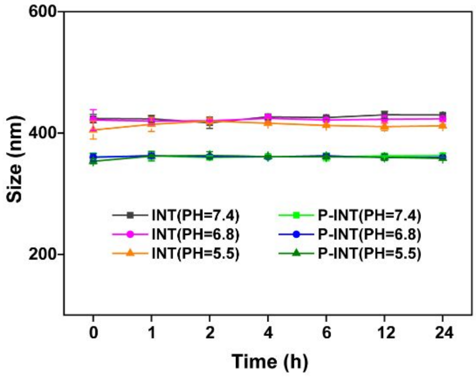

Fig. S4 Size stability of INT and P-INT at different time points under different pH conditions.

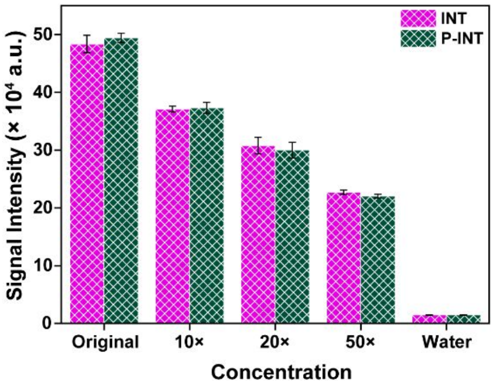

Fig. S5 The quantitative analysis of signal intensity in Figure 2d.

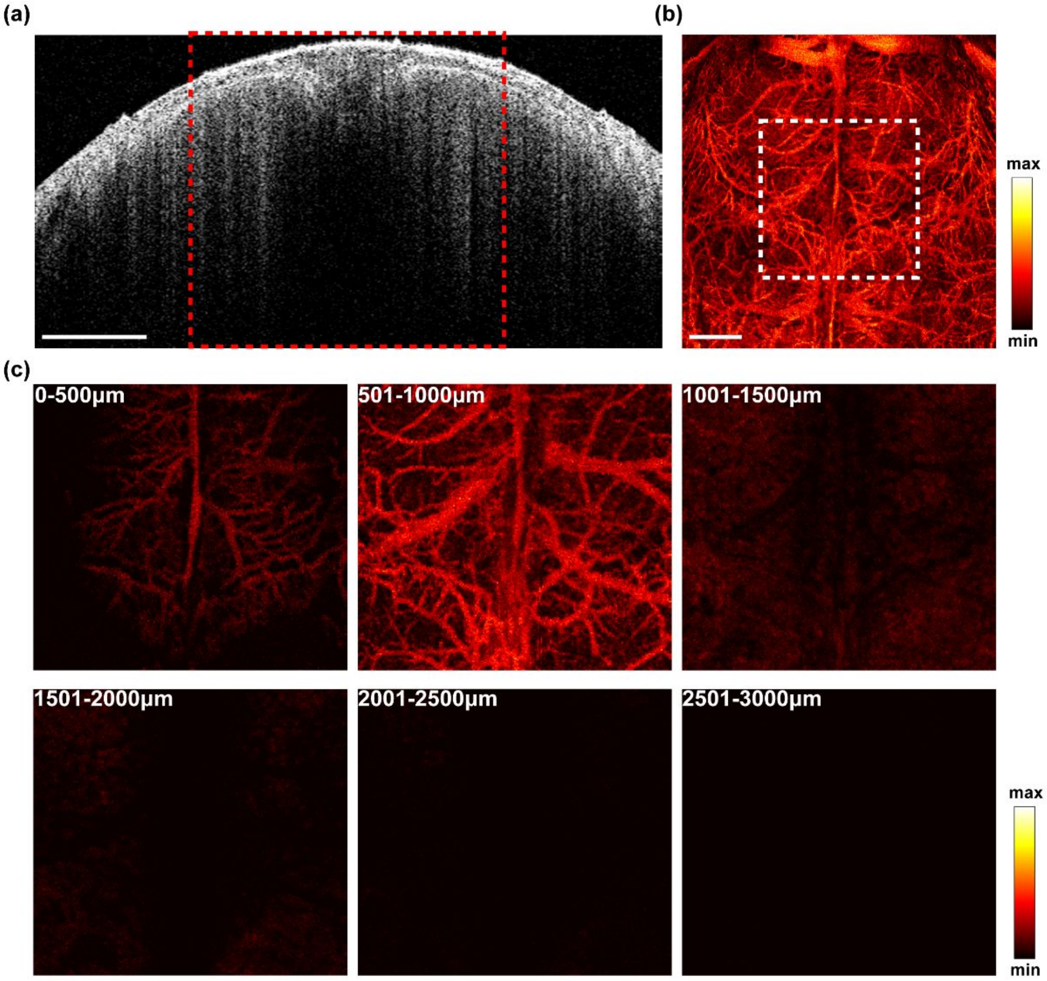

Fig. S6 The penetration depth of OCT in the NIR-II window. (a) 2D mode OCT images of mouse brain. (b) 3D mode OCT images of mouse brain. The blood flow was calculated at different depth ranges within the white dashed box area (The depth indicated by the red dashed box in (a)). (c) The significant differences in OCT results at different depths.

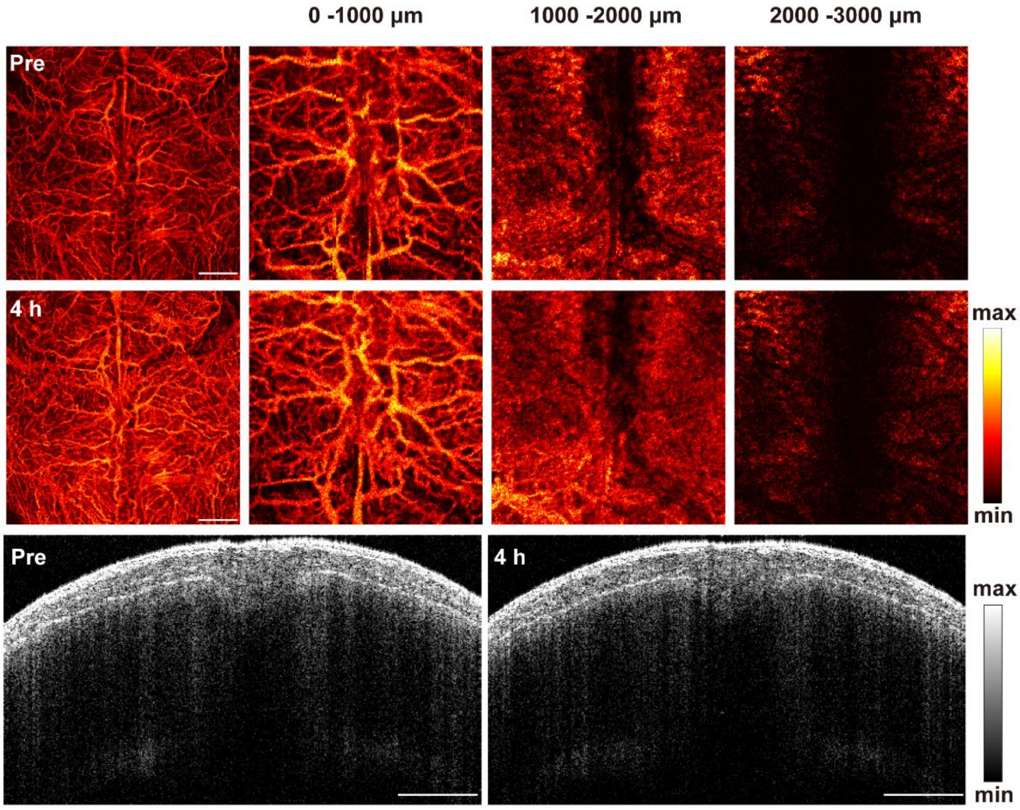

Fig. S7 OCT imaging of brain vessels at various depths after tail-vein injection of P-INT (Scale bar = 1 mm).

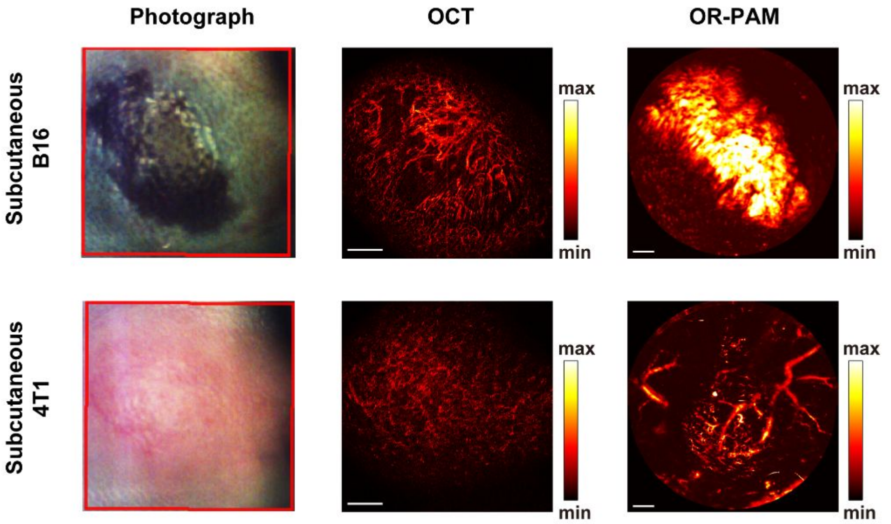

Fig. S8 The effect of melanin on OCT imaging and OR-PAM imaging. OCT and OR-PAM tumor vasculature imaging in melanin-rich B16 cell (melanoma cell lines) tumor-bearing mice and melanin-free 4T1 cell (breast cancer cell lines) tumor-bearing mice. Scale bar=1 mm.

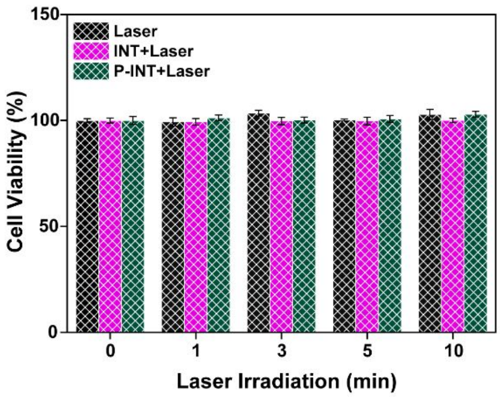

Fig. S9 The cell viability of 293T cells treated with different laser irradiation times after incubating INT and P-INT for 12 hours.

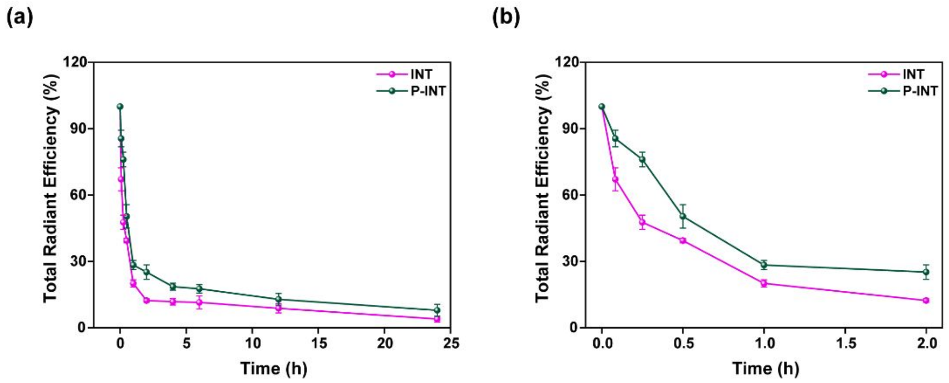

Fig. S10 Quantification of total fluorescence of blood after i.v. administration of INT and P-INT (a) and corresponding plots of enlargement (0-2 hours).

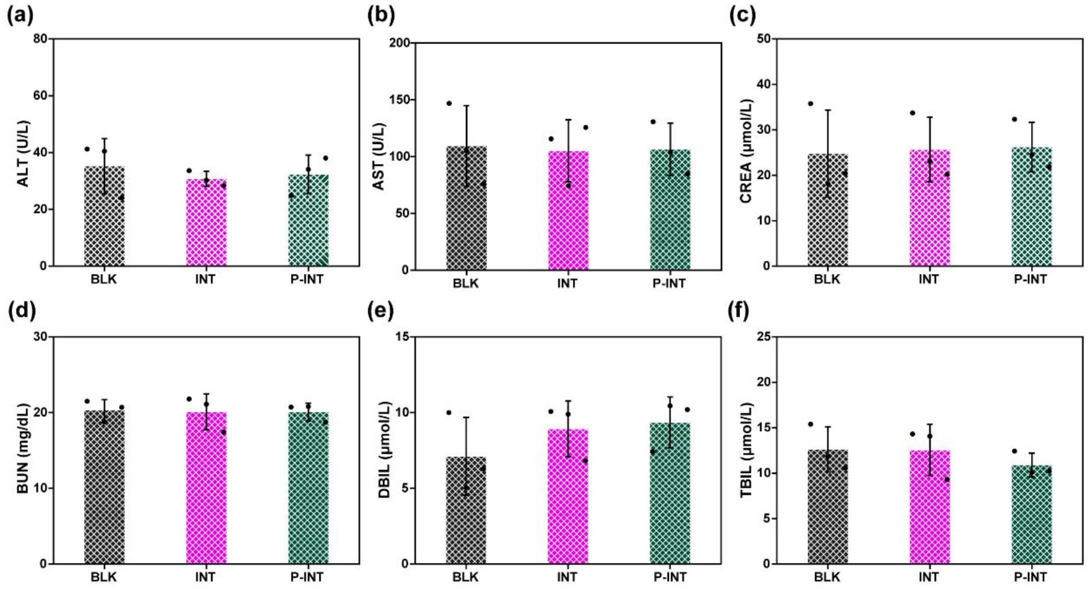

Fig. S11 (a-f) Biochemical analysis of mouse blood after 6 weeks of different treatments (injection of saline, INT and P-INT) (n=3).

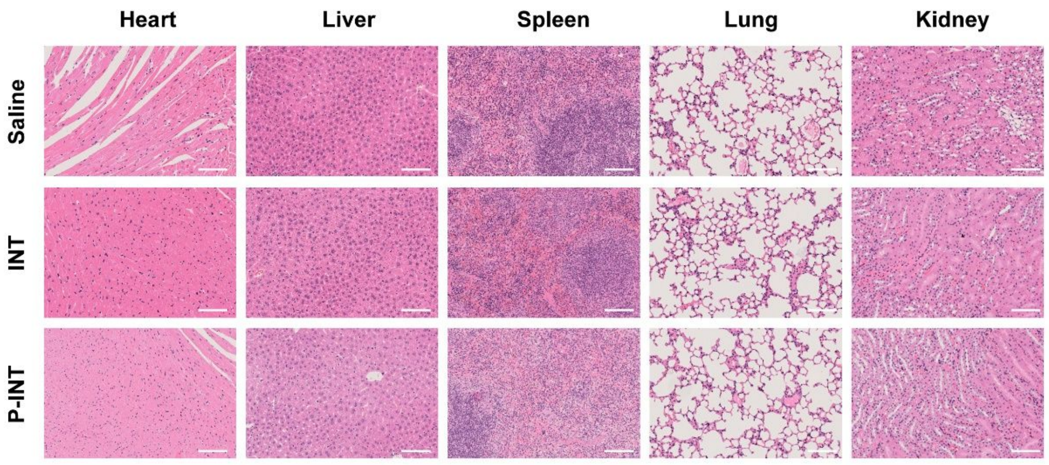

Fig. S12 Representative H&E-stained images of the major organ from mice after 6 weeks of different treatments (injection of saline, INT and P-INT) (n=3). Scale bar= 100 μm.

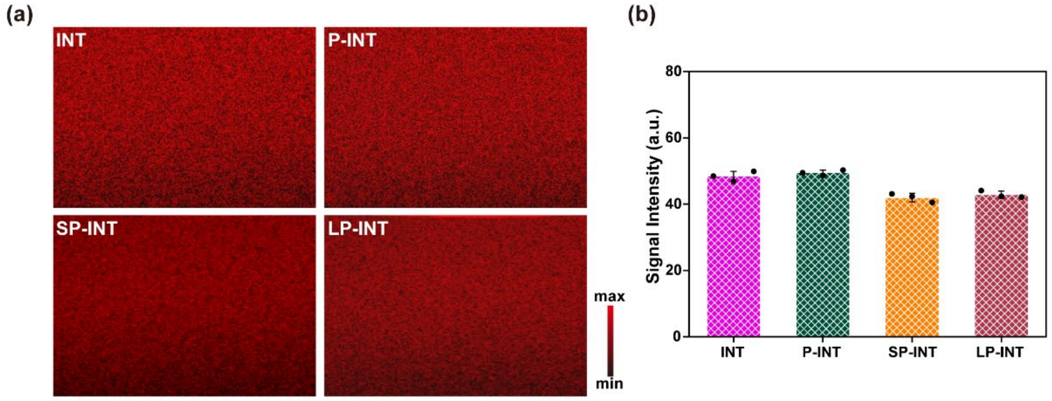

Fig. S13 Scattering properties (a) and quantitative analysis (b) of INT, P-INT, SP-INT and LP-INT.

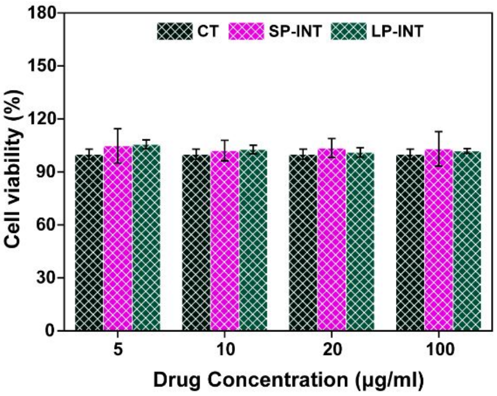

Fig. S14 The cell viability of 293T cells treated with SP-INT and LP-INT for 12 hours.

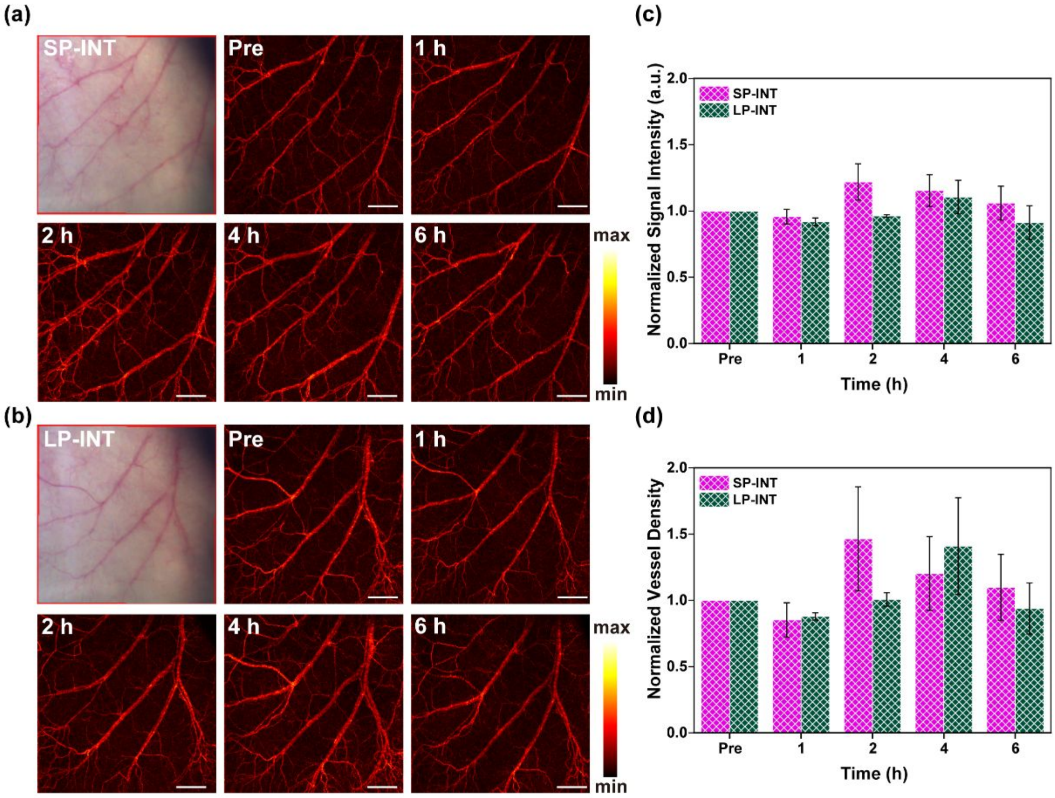

Fig. S15 OCT angiography imaging of mouse ear at different time points after tail-vein injection of SP-INT (a) and LP-INT (b). The signal intensity (c) and vessel density of mouse ear at different time points after tail vein injection of SP-INT and LP-INT. Scale bar=1 mm.
